# ‘Comfortable, safe and valued’: an analysis of the impact of COVID-19 on Hertfordshire's Community Perinatal Team

**DOI:** 10.1192/bjo.2021.808

**Published:** 2021-06-18

**Authors:** Stephanie Adeyemi, Sarah Cohen

**Affiliations:** Hertfordshire Partnership University NHS Foundation Trust

## Abstract

**Aims:**

This study aimed to assess the impact that the COVID-19 pandemic has had on the Hertfordshire Community Perinatal Team (CPT) group interventions and the innovations made.

**Background:**

The CPT is a multidisciplinary mental health service that runs three groups: Circle of Security (CoS), Emotional Coping Skills (ECS) and a peer support group - Wellbeing and Lifestyle. The service has received an increase in referrals during the COVID-19 pandemic.

**Method:**

Methods: Team member and client semi structured interviews were conducted with answers transcribed in real time and analysed. Patient clinical records were accessed via PARIS and analysed in order to identify patient demographics within each group and whether these had changed during the pandemic. Clinical outcome measures and client feedback were evaluated to see whether the change in groups is impacting their clinical effectiveness.

**Result:**

Results: Innovations made by the CPT include: groups becoming virtual, launching of the new Circle of Security Group which helps women tackle the ‘Ghosts in the Nursery’ and strengthen maternal bonds, restructuring existing groups, breakout room forums and incorporating communication platform apps such as Whatsapp. The Wellbeing and Lifestyle Group increased in size and reach (7 women from 7 areas in 2019 vs 12 women from 12 areas in 2021) with an increased retention rate (71% in 2019 vs 100% in 2021) and a decreased attrition rate (29% in 2019 to 0% in 2021). The Emotional Coping Skills group experienced similar changes (10 areas represented in 2019 vs 15 different areas in 2021) with an increased retention rate (58% in 2019 vs 100% in 2021) and decreased attrition rate (42% in 2019 vs 0% in 2021).

**Conclusion:**

The Hertfordshire Community Perinatal Team has responded to the pandemic by innovating existing groups and creating new forums; many of which will continue on even after the pandemic ceases. The groups have acted as a lifeline for women breaking up the monotony and isolation of lockdown life and providing an invaluable space for women to be heard.

